# How do cancer-sniffing dogs sort biological samples? Exploring case-control samples with non-targeted LC-Orbitrap, GC-MS, and immunochemistry methods

**DOI:** 10.1088/1752-7163/ab433a

**Published:** 2019-11-19

**Authors:** Joachim D Pleil, M Ariel Geer Wallace, James McCord, Michael C Madden, Jon Sobus, Glenn Ferguson

**Affiliations:** 1 US Environmental Protection Agency, Office of Research and Development, National Exposure Research Laboratory, 109T.W. Alexander Drive, Research Triangle Park, NC, 27709, United States of America; 2 Cancer Dogs, 25 imp del’excursion, Gatineau, Quebec, J9H6L9, Canada; 3 Author to whom any correspondence should be addressed.

**Keywords:** canine olfaction, GC-MS, high resolution MS, LC-Orbitrap, cancer screening, pre-clinical screening

## Abstract

Early identification of disease onset is regarded as an important factor for successful medical intervention. However, cancer and other long-term latency diseases are rare and may take years to manifest clinically. As such, there are no gold standards with which to immediately validate proposed preclinical screening methodologies. There is evidence that dogs can sort samples reproducibly into yes/no categories based on case-control training, but the basis of their decisions is unknown. Because dogs are sniffing air, the distinguishing chemicals must be either in the gas-phase or attached to aerosols and/or airborne particles. Recent biomonitoring research has shown how to extract and analyze semi- and non-volatile compounds from human breath in exhaled condensates and aerosols. Further research has shown that exhaled aerosols can be directly collected on standard hospital-style olefin polypropylene masks and that these masks can be used as a simple sampling scheme for canine screening. In this article, detailed liquid chromatography-high resolution mass spectrometry (LC-HR-MS) with Orbitrap instrumentation and gas chromatography-mass spectrometry (GC-MS) analyses were performed on two sets of masks sorted by consensus of a four-dog cohort as either cancer or control. Specifically, after sorting by the dogs, sample masks were cut into multiple sections and extracted for LC-MS and GC-MS non-targeted analyses. Extracts were also analyzed for human cytokines, confirming the presence of human aerosol content above levels in blank masks. In preliminary evaluations, 345 and 44 high quality chemical features were detected by LC-MS and GC-MS analyses, respectively. These features were used to develop provisional orthogonal projection to latent structures-discriminant analysis (OPLS-DA) models to determine if the samples classified as cancer (case) or non-cancer (control) by the dogs could be separated into the same groups using analytical instrumentation. While the OPLS-DA model for the LC-HR-MS data was able to separate the two groups with statistical significance, although weak explanatory power, the GC-MS model was not found to be significant. These results suggest that the dogs may rely on the less volatile compounds from breath aerosol that were analyzed by LC-HR-MS than the more volatile compounds observed by GC-MS to sort mask samples into groups. These results provide justification for more expansive studies in the future that aim to characterize specific chemical features, and the role(s) of these features in maintaining homeostatic biological processes.

## Introduction

### Background

A crucial factor in public/environmental health and medical research is assessing pre-clinical health state in individuals. The earlier an intervention measure is invoked, the better the ultimate outcome will be (Dietrich et al 1992, Gasparri et al 2018, Januszewicz and Fitzgerald 2019, Wild et al 2019). Clinical biomarker screening efforts seek markers of disease at the individual level, while public health research efforts examine various exposure and health markers in populations of interest (Baker 2000, Pepe et al 2001, Srivastava and Gopal-Srivastava 2002). Many different approaches have been developed for health state monitoring and prediction of future disease, most based on some form of biomonitoring of blood, breath or urine (Hunter 2005, Wild 2005, Laxman et al 2008, Rappaport and Smith 2010, Allegra et al 2012, Laurenco and Turner 2014, Hayes et al 2016). These studies rely on group comparison strategies wherein known healthy and sick cohorts are contrasted, or on longitudinal studies where the deviation of a biomarker pattern is monitored with respect to baseline patterns (Rutjes et al 2005, Pleil et al 2011b, Morozov et al 2017, Beccaria et al 2018, Steckling et al 2018). Although laboratory instrumentation methods using gas- and liquid-chromatography-mass spectrometry (GC-MS and LC-MS, respectively) dominate the literature, there are now a number of organizations using specially trained dogs to sort biological samples for follow-up and future diagnostics. Both approaches have their proponents and detractors, as discussed in recent literature (Sawyers 2008, Hackner et al 2016, Ferguson 2017, Hackner et al 2017).

In this article, we present a pilot-level exploration of breath sample screening via canine olfaction and analytical chemistry. The objectives are to: (1) collect breath mask samples that have been unambiguously classified by cancer-sniffing dogs as belonging to a ‘case’or ‘control’ group; (2) measure and compare targeted inflammatory cytokine levels within mask extracts to provide evidence of human breath aerosol capture; (3) offer evidence for the presence of chemical features within mask extracts using multiple non-targeted analysis (NTA) methods (i.e. GC-MS and LC-MS); and (4) differentiate chemical features across groups using supervised statistical modeling. As this is a pilot study with limited sample size, efforts are not made to confirm the presence of specific chemical analytes, or to relate the presence of those analytes to biological pathways linked to cancer or other health endpoints. Future studies may focus on unambiguous chemical identification, biological pathway interrogation, and application of statistical models towards sample classification in pursuit of biomarkers or complete elucidation of the canine recognition system. The following section provides background information on concepts that are foundational to this pilot-scale exploration.

### Environmental and health screening concepts

Regardless of the ultimate methodology used for preclinical disease screening, there are certain concepts that must first be considered. These are:

- Human exposome
- Non-targeted analysis
- Targeted analysis
- Case-control study
- Preclinical health state definition
- Caveats

The *human exposome* was originally defined as all exposures, for an individual, from conception onwards (Wild 2005); example exposures that can be measured within a biological system include exogenous (environmental) chemicals, metabolites, and the wide range of endogenous (life process) compounds, including proteins, protein and DNA adducts, chemokines, and reactive oxygen species (Miller and Jones 2013, Dennis et al 2016). The underlying concept for preclinical disease assessment is that there are unique changes in the exposome (and the related metabolome) that can be predictive or indicative of health state changes (Wild 2005, Rappaport 2011, Pleil 2012, 2016, LaKind et al 2014, Stiegel et al 2017).

NTA, or ‘discovery’ analysis is a primary analytical tool for assessing what parts of the exposome might be probative in linking with a burgeoning disease state. The analyses are performed without preconception, and the methodology is broad; the general idea is to test for as many chemicals as possible in biological media (blood, breath urine, etc) and derive descriptive statistical models from the resulting data. High-resolution (HR-MS) instruments, either with time-of-flight or Orbitrap mass analyzers, are the workhorse tools for this approach because HR-MS provides added dimensions for identifying unknown features via accurate mass measurements, isotopic prediction of chemical formula, and structural elucidation via fragmentation; we have previously discussed specific methods here (Pleil and Isaacs 2016, Pleil et al 2018). The lack of a pre-determined analyte list allows the data to be queried at leisure for any compounds or features determined to be of interest after the fact (Pleil and Stiegel 2013, Rager et al 2016, Milman and Zhurkovich 2017, Newton et al 2018, Sobus et al 2018). However, the complexity of the data often requires complex software approaches for identification of metabolites and modeling of the underlying systems (Yi et al 2016).

The compound identification schemes used herein go well beyond the ‘library search only’ approach. US EPA is in the forefront of developing methods and databases for NTA. Accordingly, this work is based on internal procedures and workflows (Sobus et al 2018, Wallace et al 2019), as well as the open source US EPA DSSTox database found at: (https://comptox.epa.gov/dashboard/chemical_lists) to help understand the results of non-targeted results (Pleil and Williams 2019).

*Targeted analysis* is a standard laboratory analysis in which only specific compounds of interest (selected *a priori*) are sought. Targeted analyses are generally performed with selected ion mass spectrometry or with MS/MS (e.g. triple-quadrupole) instruments. The advantage here is that the methodology is initially optimized for the compounds of interest resulting in better specificity (identification) and highly sensitive quantitation (Lu et al 2008, Gillette and Carr 2013, Wallace et al 2017, 2019).

*Case-control* studies are one of the primary contrast methods for identifying potentially probative biomarkers. The concept is to compare matched groups of subjects that differ only in their diagnosed disease state. Comparisons are contemporary and by group, and as such, not paired. The main challenges are in choosing the correct groups for comparison and having sufficient subjects to avoid over-fitting models (Schlesselman1982, Pleil et al 2012).

*Pre-clinical health state* refers to a condition somewhere on the path between a relatively healthy and unambiguously diagnosed disease state. Identifying individuals with preclinical indications of disease is valuable because early intervention is beneficial to slow or reverse disease progression. Identification is difficult, however, because there is generally no medical gold standard for preclinical assessment (Bjorner et al 2005, Loscalzo et al 2007).

*Caveats* comprise the cautions associated with conducting screening efforts for markers of disease or exposure. There are four basic assumptions that need to be evaluated:

- The first assumption is that the methodology (dog or analytical chemistry) can actually detect the probative markers. Chemical signals of biological differences may be weak and obscured by chemical background, or not conducive to a particular analysis approach (e.g. specific chromatographic and ionization methods).
- The second assumption is that a singular disease biomarker is the same for clinical and preclinical conditions; that is, a marker differentiating case and control groups is the same regardless of the extent of disease progression.
- The third assumption is that an appropriate ‘control’ group, free of disease state, can be obtained to create a classification model; this may be difficult, as a preclinical disease state is not strictly medically diagnosed and so the control group could include preclinical cases.
- The fourth assumption is that all individuals express the same biomarkers in response to development of the disease state, and that responses are not masked by population or individual level variations. Inter-individual-variability of metabolism is possibly affected by ethnicity, gender, age, health state, diet, medications, and genetic/phenotypic differences (Nicolas et al 2009, Lewis et al 2018).

### Classifying preclinical samples: the screening approach

When investigating disease in the preclinical state, validation of ultimate disease progression to assign case-control status requires waiting for the prognosis, which can be inefficient. Latency periods for cancers are on the order of 5–50 years, and the incidence rate for all cancers combined is ~0.44% (www.cancer.gov/about-cancer/understanding/statistics). For specific cancers the incidence rate is even lower, requiring thousands of subjects to field a statistically valid study. This makes direct recruitment for long-term studies unwieldy. High risk groups can be used instead, but these may not be representative of the whole population.

Another approach to acquiring suitable sample sets is to incorporate retrospective case-control epidemiological methods. The concept is to assess current health state of a population for which archived biological specimens are accessible, possibly for unrelated reasons, and sort them into two equal sized case-control groups. Now, the relevant samples can be pulled from the archives and compared with the full knowledge of the outcome. This approach avoids waiting many years for a rare outcome but relies on large sets of properly stored specimens. These sets are sometimes available as part of other screening or testing regimes, such as dried blood spots (Guthrie Cards) from newborn children (Funk et al 2008, Stove et al 2012, Funk et al 2015).

The goal of developing biomarker methods for predicting cancer and other diseases is to intervene well before the occurrence of irreversible symptoms and damage. Certainly, individuals with environmental, occupational, and family history risk factors are of primary concern for studies described above, and markers of risk can be equally as instructive for preclinical assessment as biological indicators of disease. For example, subjects with historical asbestos exposures can be tested regularly with imaging for physiological changes in the lungs and for tumors. More recently, a blood test for the biomarker protein osteopontin has been applied to predict early cases before physiological changes occur (Rangaswami et al 2006). Breath tests based on exposures to volatile organic compounds (VOCs) related to chemical patterns indicating lung cancer have been proposed for smokers, and tests of urinary metabolites of exposures and proteins are used to infer associations with kidney and bladder cancer in workers exposed to polycyclic aromatic hydrocarbons (Jongeneelen et al 1988, Phillips et al 1999, Pleil and Stiegel 2013). In all these methods, biomarkers were initially explored using case-control targeted or discovery analyses.

### Canine olfaction and chemical laboratory strategies

Preclinical classification is based on sorting biological specimens into yes/no groups, based on some underlying measurement(s). From a philosophical standpoint, sorting by trained dogs is a holistic, opaque system, where detection and sorting occur within the dog’s brain. Analytical instrumentation is more transparent process in that ultimately the classification can be examined to reveal probative chemicals for the sorting process; these chemicals could be further linked to biochemical pathways. Both strategies have respective challenges and advantages (Pleil and Giese 2017).

*Canine olfaction*, or dog sniffing, requires teaching dogs how to generalize sorting beyond their familiar training sets. Dogs can sort dozens of samples (including blind replicates and known control samples) in a few minutes. Presumably, the dogs use a pattern recognition approach based on odorant binding at more than 220 million olfactory receptors in their nasal cavities (Jenkins et al 2018). The problem with canine olfaction arises when the dogs are exposed to completely different samples. Many ‘double-blinded’ studies disparaging dogs’ abilities to sort samples fall prey to this training failure. For dogs to become expert in sorting, they need to be exposed to many hundreds of different samples randomized over different sessions, with new known case-control samples inserted periodically. Their training results need to be tracked over time to assess if they are consistent, and whether they accurately pick out known samples. Dogs who do not perform successfully need to be removed from the rotation. These issues were described by Elliker et al (2014) who found that the dogs may not generalize patterns until they have exceeded their olfactory memory capacity. According to CancerDogs, samples need to be assessed by multiple dogs (ideally 4–5), and in more than one session to confirm a consensus positive. Optimally, training and working are combined in an ongoing process using an extensive sample library so that each session of new real-world samples includes known cases and controls to test the dogs’ accuracy in sorting.

Some challenges to canine olfaction are that dogs can only work for short periods of time; they are prone to distraction, boredom, fatigue, hunger, biological needs, etc Their performance is driven by a reward system of small treats, which makes false positives a distinct possibility (Hackner and Pleil 2017). Furthermore, running a dog-based laboratory requires special handling skills and a 24/7 kennel operation. From an analytical perspective, understanding their behavior is further complicated by the wide range of analytes (gases, semi-volatiles, aerosols, particles) contributing to the pattern; this is an advantage in terms of breadth of analysis, but makes replication difficult.

*Chemical laboratory analysis* can be carefully controlled and documented. Instruments can be operated with little downtime, and once methods are established, can be automated for repetitive analyses. Initial, detailed chemical analysis can require high levels of technical expertise and lengthy instrument run times (generally tens-of-minutes). LC-MS instruments, especially high resolution mass spectrometers, are expensive and require expert maintenance. Furthermore, data interpretation, especially for non-targeted analyses, may initially take many hours per sample until sufficient statistical results are accumulated to develop a streamlined targeted approach. Finally, just as in training of dogs, it is important to have a sufficient number of known samples to develop the overall patterns in the face of human variability.

### Implementing a hybrid study design

As mentioned above, there are cautions for some of the underlying assumptions when exploring individual biomarkers or complex classification models via a NTA approach. The motivation for this work is to use two disparate systems, canine olfaction and laboratory instrumentation, to explore the consensus classification of preclinical specimens and improve the understanding of the mechanism of classification.

**Figure 1.**
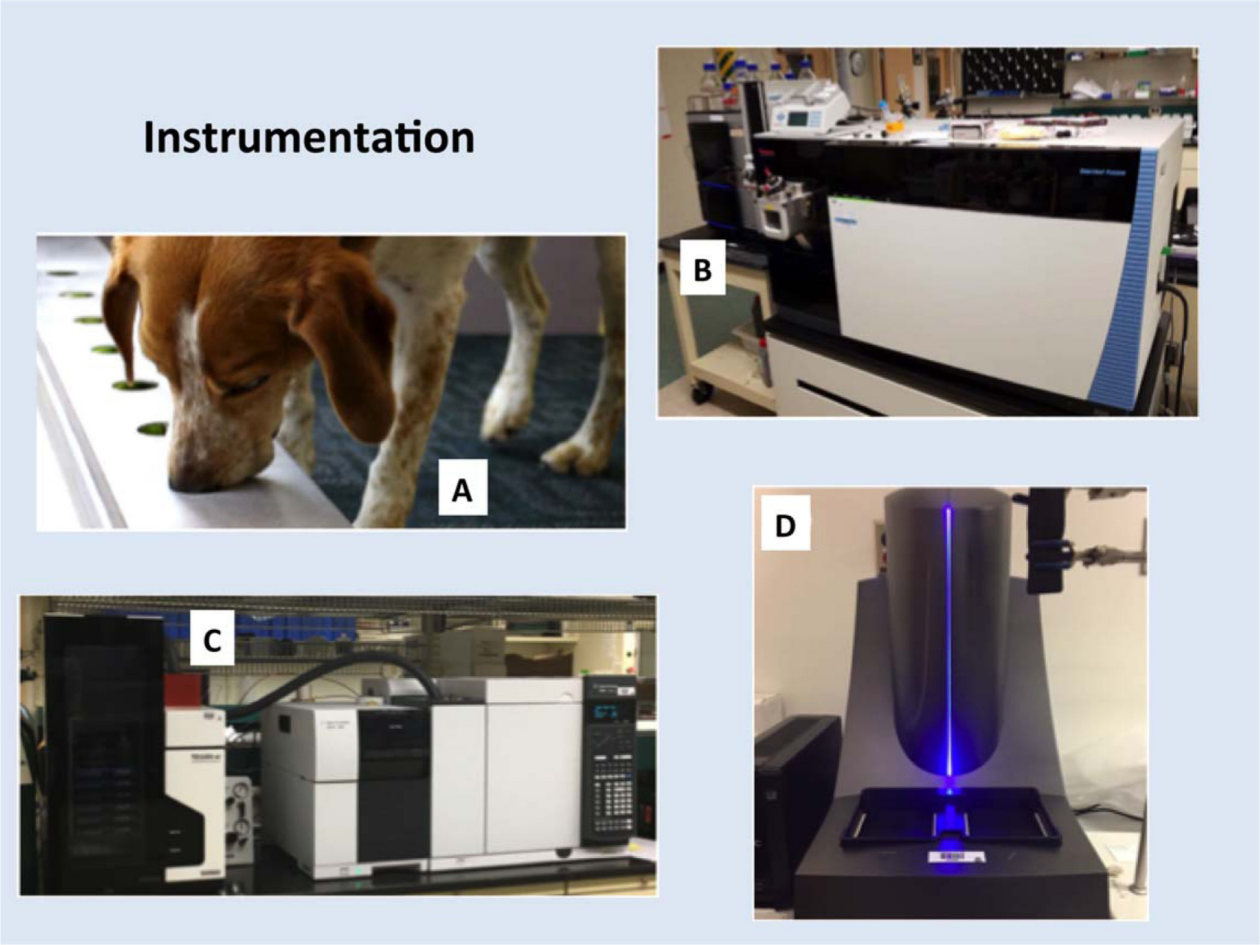
Analytical tools for assaying aerosol samples. (A) Trained dog; (B) LC-Orbitrap HR-MS; (C) thermal desorption GC-MS; (D) immunochemistry platform.

In this article, we investigate a set of biological specimens drawn from an ongoing program of canine olfaction evaluation and study them in detail using laboratory instrumentation. Given the ability of dogs to sort samples into groups (regardless of accuracy), we investigated whether standard hospital masks that are used for canine diagnostics can capture probative human aerosols and whether analytical chemistry can be used to uncover chemical signatures that likely influence dogs’ decisions in selecting potential pre-clinical cancer samples. Figure 1 shows the four approaches that were used in this investigation: trained dogs, liquid chromatography-high resolution mass spectrometry (LC-HR-MS), thermal desorption GC-MS, and immunochemistry. This is a first effort to assess the potential value of combining the two disparate diagnostic methods. This demonstration will pave the way towards more comprehensive studies using an independent cross-over design of greater numbers of samples.

## Methods

### Biological specimens

This investigation was approved under Human Studies Research Review request HSR-001023 by US Environmental Protection Agency (EPA).The samples were received as anonymous biological specimens from CancerDogs in Quebec, Canada, http://cancerdogs.ca/. The specimens included two sets of ten hospital-style olefin polypropylene disposable masks (Medline, Northfield, IL) that were worn by humans and sorted into categories of cancer (i.e. case) and non-cancer (i.e. control) by cancer-sniffing trained dogs at CancerDogs. The samples were kept as blinded as possible with respect to meta-data to avoid selection bias. The subjects consisted of off-duty fire fighters in US fire departments that collaborated with CancerDogs for early cancer screening of presumably healthy individuals (no existing cancer diagnoses). The samples were collected remotely by subjects who wore the masks for ~10 min during normal activity and then mailed the masks to CancerDogs in sealed pouches. The masks were then screened by four dogs in individual sessions, and all four dogs unanimously agreed on the classification of these samples as either case or control.

Selected masks were provided to the US EPA laboratory after unambiguous consensus class assignment by the four dogs. Two blank olefin polypropylene masks of the same style and material used for sampling were also provided by CancerDogs. These masks were not worn by humans or assessed by the dogs but were processed along with the samples and used as instrument blanks. This mask-based sampling strategy represents a practical and non-invasive method for collecting human specimens; it is not designed to collect VOCs, but is presumed to capture semi-volatile and non-volatile species from the aerosols fraction of breath. This sampling strategy has been described in the literature (Pleil and Giese 2017, Pleil et al 2018) and the extraction of breath aerosols from both hard and soft surfaces has been described in Wallace et al (2019).

### Sample extraction

Four 2 × 2 in (5 × 5 cm) sections were cut from each mask and immediately placed into 15 ml plastic Falcon tubes. Two samples from each mask were prepared for LC- and GC-MS analysis and two for immunochemical (ELISA) analysis. The sections earmarked for MS analysis were soaked in 5.5 ml methanol, centrifuged, sonicated for 30 min, vortexed for 1 min, centrifuged, and the resulting extracts were transferred to new vials. The extracts were centrifuged again and concentrated using a steady stream of high-purity N_2_ until the extract volume was reduced to less than 1 ml. The sample extracts were stored at 4 °C prior to additional preparation for LC- and GC-MS analyses.

The remaining two mask samples for ELISA analysis were soaked in 2 ml phosphate buffered saline with 0.05% bovine serum albumin and placed on a rotator at 4 °C overnight. The samples were then vortexed 30 s, centrifuged at 500 × g at 10 °C for 5 min, and the supernatants were removed and stored in polypropylene tubes at 4 °C. Samples were analyzed within 7 d. Two unused laboratory blanks were similarly processed using the LC-MS and ELISA preparation procedures.

### Sample processing and analysis: immunochemistry

Immunochemistry aliquot samples were analyzed using the MSD V-plex kit human proinflammatory panel 1 containing the following ten cytokines: IFN-*γ*, IL-10, IL-12p70, IL-13, IL-1*β*, IL-2, IL-4, IL-5, IL-8, TNF-*α*. Aliquots (50 *μ*l) were injected directly into 96-well plates. Standards and samples were analyzed in duplicate using a MesoScale Discovery Quickplex 120 instrument (Mesoscale Discovery, LLC, Rockville MD, USA). This instrumentation has demonstrated sensitivities ranging from 0.03 to 1.37 pg ml^−1^ in EBC depending on compound and human subject (Stiegel et al 2015). Recall that aerosols were reconstituted into a 2 ml liquid (phosphate buffered saline) from 2 × 2 in sections of masks and so should be analogous to EBC. These procedures have been developed and used for a number of applications at US EPA for longitudinal exposure studies (e.g. Stiegel et al 2015, 2016, 2017) and the overall concept of how immunochemistry fits with other methods of breath analysis has been reviewed in Wallace and Pleil (2018).

### Sample processing and analysis: LC-HR-MS

Sub-aliquots of the final 1 ml volume MS-designated sample replicates and two mask blanks (in methanol) were diluted with 2 mM ammonium formate. The diluted samples were separated on a Vanquish UPLC system equipped with an Accucore Vanquish C18 + column (100 × 2.1 mm, 1.5 *μ*m particle diameter) coupled to an Orbitrap Fusion for analysis (ThermoFisher Scientific, Waltham, MA, USA). MS data were collected with both positive and negative electrospray ionization in data-dependent MS/MS mode with a stepped HCD collision energy. Additional details regarding instrumental analysis can be found in section 1 of the supplemental information, which is available online at stacks.iop.org/JBR/14/016006/mmedia.

### Sample processing and analysis: GC-MS

Sub-aliquots of the 1 ml MS sample extracts, two mask blanks (in methanol), and one methanol blank were prepared for GC-MS analysis. Briefly, 1 *μ*l sample aliquots (or methanol) were spiked onto the screen of Carbograph 2TD/1TD sorbent tubes (Markes International, Gold River, CA, USA) using a 10 *μ*l gas-tight syringe, and sorbent tubes were placed into a loading dock with a 50 ml min^−1^ stream of research grade helium for 1 min at room temperature.

Samples were analyzed on the same day that they were loaded using a Markes TD 100-xr automated thermal desorber (Markes International, Gold River, CA, USA) coupled with an Agilent 7890B/5977B GC-MS (Santa Clara, CA, USA). Samples were run using scan detection mode from 35–300 *m/z* with unit Da resolution. Thermal desorption was achieved using a 5 min prepurge, 20 ml min^−1^ standby split, and 10 min tube desorption at 375 °C. A 30 m Restek Rtx-VMS column (part no. 1547218) with a 0.25 mm ID and 1.4 *μ*m film thickness was used for chromatographic separation with research grade helium as the carrier gas at a flow rate of 2 ml min^−1^. The oven temperature was held at 30 °C for 3 min, ramped to 150 °C at 8 °C min^−1^, and ramped to 240 °C at 36 °C min^−1^ for a 5 min hold. The MS source and quad were set to 230 °C and 150 °C, respectively.

We note that reproducibility of GC-MS analysis of polar VOCs such as aldehydes, ketones and alcohols may be affected by co-collected water vapor from breath-based samples, however, we have successfully applied methodologies for adsorbent transfer of these compounds for chromatographic analysis (Pleil et al 2008, Hubbard et al 2009, O’Lenick et al 2019).

### Data processing: immunochemistry

Cytokine concentrations were reported in pg/mL, and the concentrations obtained for replicate masks from the same individual were averaged prior to graphing or statistical analyses. For statistical analyses, the data were natural log-transformed (log_e_), and one-tailed t-tests were conducted to evaluate statistically significant elevations between the blank and control samples as well as between the blank and case samples. The concentrations measured for TNF-*α* were below detection limits and are not reported herein. Sample post-processing and low-level imputation were conducted under protocols outlined in Stiegel et al (2015).

### Data processing: LC-MS

Raw MS data were processed using Compound Discoverer 2.1 software (Thermo Scientific) for retention time correction, peak picking, feature alignment, molecular formula generation, and database searching (based on monoisotopic mass, predicted formula, and MS/MS spectra) against mzCloud and ChemSpider using the Environmental Unknown ID workflow with default settings. Missing features were imputed with statistical noise using the ‘fill peaks’ option. Approximately 2000 combined features were detected across all samples and modes and filtered to remove spurious and background peaks. Features were removed if the peak area abundance in at least one sample was not >5× the signal in the blank mask or if the feature was not detected in all technical replicate analyses. Feature quality (accurate alignment, spectral picking, and integration) was checked via manual examination of the LC peaks and MS spectra. Duplicate features or those deemed to be instrumental artifacts were removed. Ultimately, 345 ‘high quality’ features were exported for further statistical modeling in R. Compound assignments were prescribed by Compound Discoverer as the top scoring database hit based on accuracy of formula match and number of fragment ions described by the precursor formula.

### Data processing: GC-MS

GC-MS non-targeted analyses were performed similarly to the LC-MS analyses with the exception that the resulting MS data were at 1 Da resolution, thus restricting the overall specificity. Raw GC-MS data were exported to Agilent Profinder for deconvolution, peak picking and alignment. Peaks were extracted at 1 Da resolution and deconvoluted to produce chemical spectra, which were aligned across samples and integrated. Similarly to the LC-MS data, chemical features were removed if they did not pass an abundance difference compared to blank masks (>5× abundance in blanks). Tentative identifications were assigned using Agilent Unknowns Analysis software through matching to the NIST 14 MS library. Subsequently, results were individually curated to remove unlikely identifications and exported for further modeling.

### Orthogonal projection to latent structures-discriminant analysis (OPLS-DA)

MS feature sets were normalized using robust quantile normalization (*preprocessCore:* Bolstad 2013) to attempt to account for inter-individual biological variability and log-transformed, mean centered, and z-scaled for modeling. Sample groups were assigned based on the case/control status as assigned by the dog sniffing screen. Sample classification models were built using a multivariate OPLS-DA approach implemented in the R package *ropls* (Thévenot 2016). PLS-DA is similar to principal component analysis in that it projects measured variables onto a reduced number of latent variables for model creation. OPLS-DA extends this approach by maximizing the between-class variance in a single predictive component, with the remainder of the model composed of non-predictive, orthogonal components. This improves the interpretability of the model without altering its classification effectiveness (Trygg and Wold 2002). Both PLS-DA and OPLS-DA are categorization models—in this case designed to mimic the assignment provided by the dog sniffing. Decomposition of the classification model should reveal important chemical signals for conducting the class assignment.

LC and GC feature matrices were used to construct two classification models in *ropls*. Exploratory PLS models were constructed to optimize the number of model components, and an OPLS model was constructed with one predictive component and sevenfold cross validation. Model significance was calculated by comparison with a population of null models constructed from 1000 random permutations of the sample labels. This is necessary because OPLS-DA models can easily produce apparently well-separated models from noise if the number of features exceeds the number of observations, as is the case here (Wehrens 2011).

## Results and discussion

The cytokine analyses confirmed that the mask samples represented human exhaled aerosols and that there is interpersonal variability, as expected. For LC-HR-MS data, there were 345 distinct features that stood out sufficiently above blank and instrument controls and appeared in both sample groups (see supplemental table S1). For GC-MS data, there were only 44 distinct features that were used (see supplemental table S2). The OPLS-DA modeling was able to separate sample groups using chemical data derived from LC-HR-MS, but GC-MS non-targeted results were not statistically significant. Lists of the high quality features used for LC and GC modeling are provided in tables S1 and S2.

### Immunochemistry results

The main purpose for the immunochemistry analyses was to determine if the mask samples contain endogenous human organic compounds based on highly specific analysis for human cytokines. Inflammatory cytokine levels are not known to be indicative of a pre-clinical cancer state, and therefore were not expected to determine how the dogs sorted samples, although it is possible cytokines could contribute to observed differences.

**Figure 2.**
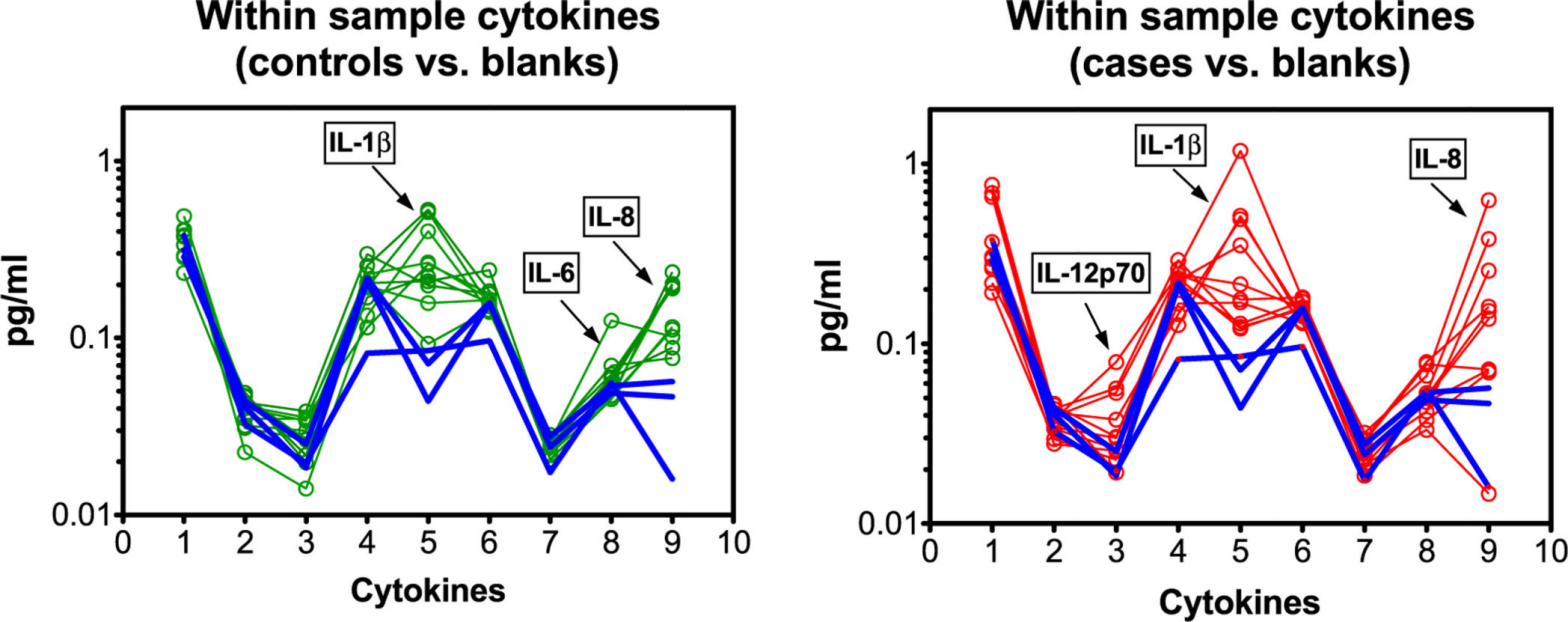
Comparisons of cytokines (*x*-axis) and within-sample pattern variability (*y*-axis) with respect to blanks; each individual sample is connected with lines. Cytokines are numbered in order listed above. The left panel shows the comparison of controls (green) versus blanks (blue) and the right panel shows cases (red) versus blanks (blue). IL-1*β*, IL-6, and IL-8 appear higher than blanks for controls; IL-12p70, IL-1*β*, and IL-8 appear higher than blanks for cases.

Results for nine human cytokines (IFN-*γ*, IL-10, IL-12p70, IL-13, IL-1*β*, IL-2, IL-4, IL-5, IL-8) showed detectible levels in mask samples sorted by dogs, and in ‘blank’ mask samples that were handled, but not used for collecting breath aerosol. Handling of masks by individuals not wearing gloves has been previously shown to contribute to cytokine levels in unpublished laboratory analyses, presumably due to contact with skin. However, figure 2 shows that a number of cytokines were present at levels above those of the blank masks, indicating that some cytokines originated from breath aerosol. Figure 2 shows the within- and between-sample pattern variability for cytokines numbered in order from left to right as in the list above. The left panel shows the comparison of controls (green) versus blanks (blue) and the right panel shows cases (red) versus blanks (blue). The cytokine TNF-*α* (#10) showed no detectible response above the limit of detection and was excluded from analyses. The patterns of cytokine concentrations within a sample were compared in figure 2 to detect cytokines that did not conform to the overall patterns of the control and case groups (such as IL-1*β*).

**Figure 3.**
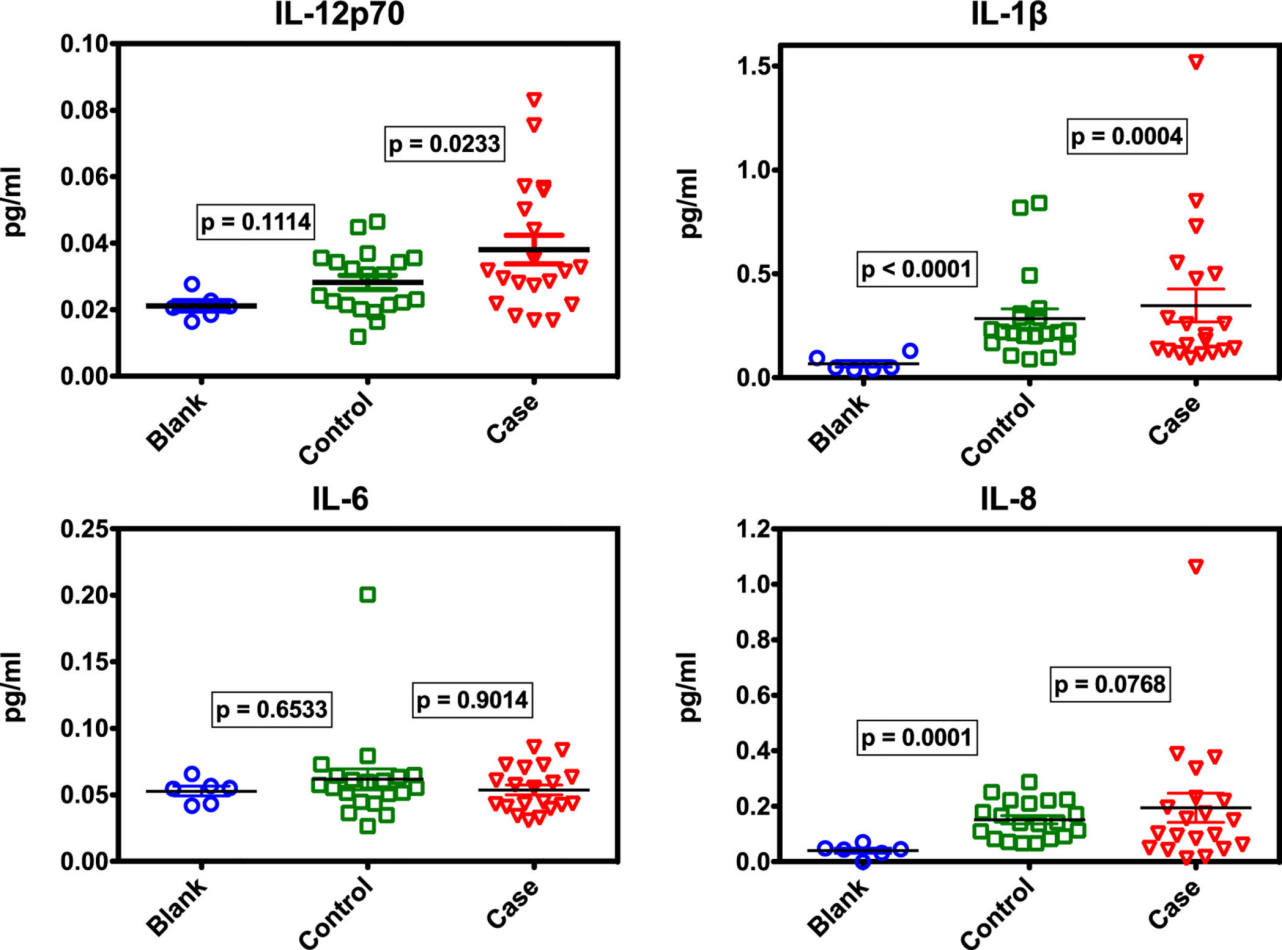
Comparison of select cytokines (those listed in figure 2) across sample groups. *P*-values are from t-tests of natural log-transformed (log_e_) concentrations of sample versus blanks. Statistically, IL-12p70 cases, IL-1β controls and cases, and IL-8 controls are all different from blanks (*p* < 0.05), indicating that human cytokines in breath are collected on masks.

These results show that some human cytokines are present in the mask samples beyond those from background (no breath) handling. Detailed results for IL-12p70, IL-1*β*, IL-6, and IL-8 are shown in scatterplots in figure 3. The *p*-values from log-space t-tests for blank versus control, and blank versus case are shown on the plots. Of interest is that the plots of IL-6 values in the left panel of figure 3 show little difference across groups. The remainder, IL-12p70, IL-1*β*, and IL-8 indicate statistically significant differences in one or more sample groups compared to the blank masks, confirming that there are indeed human cytokines captured from breath. The apparent contribution to cytokine levels from handling masks without gloves made the cytokine analysis more difficult to interpret. This could be alleviated in future studies by having all participants wear gloves while performing breath sampling to reduce background contamination.

### Summary of immunochemistry results

Measurement of human cytokines on mask samples, at levels appreciably higher than on mask blanks, confirms that breath aerosols are collected on, and can be recovered from, the mask substrate material. This implies that the other semi- and non-volatile compounds found in LC-HR-MS and GC-MS analyses that are above background levels in blank masks are also likely from breath aerosols. The cytokines, as a group, do not appear to be differentially expressed overall between the control and case groups; however, some individual cytokines (e.g. IL-12p70) might, in the future, be considered as probative markers.

### LC-HR-MS results

Identifications of LC-HR-MS compounds were based on default settings in Compounds Discoverer 2.1 for formula prediction and searching against ChemSpider (for putative chemical assignments). A total of 106 compounds out of 345 were assigned a chemical name. Assignment confidence was based on the scale of Schymanski et al (2014) (supplemental table S1).

### Feature-based OPLS-DA analysis

**Figure 4.**
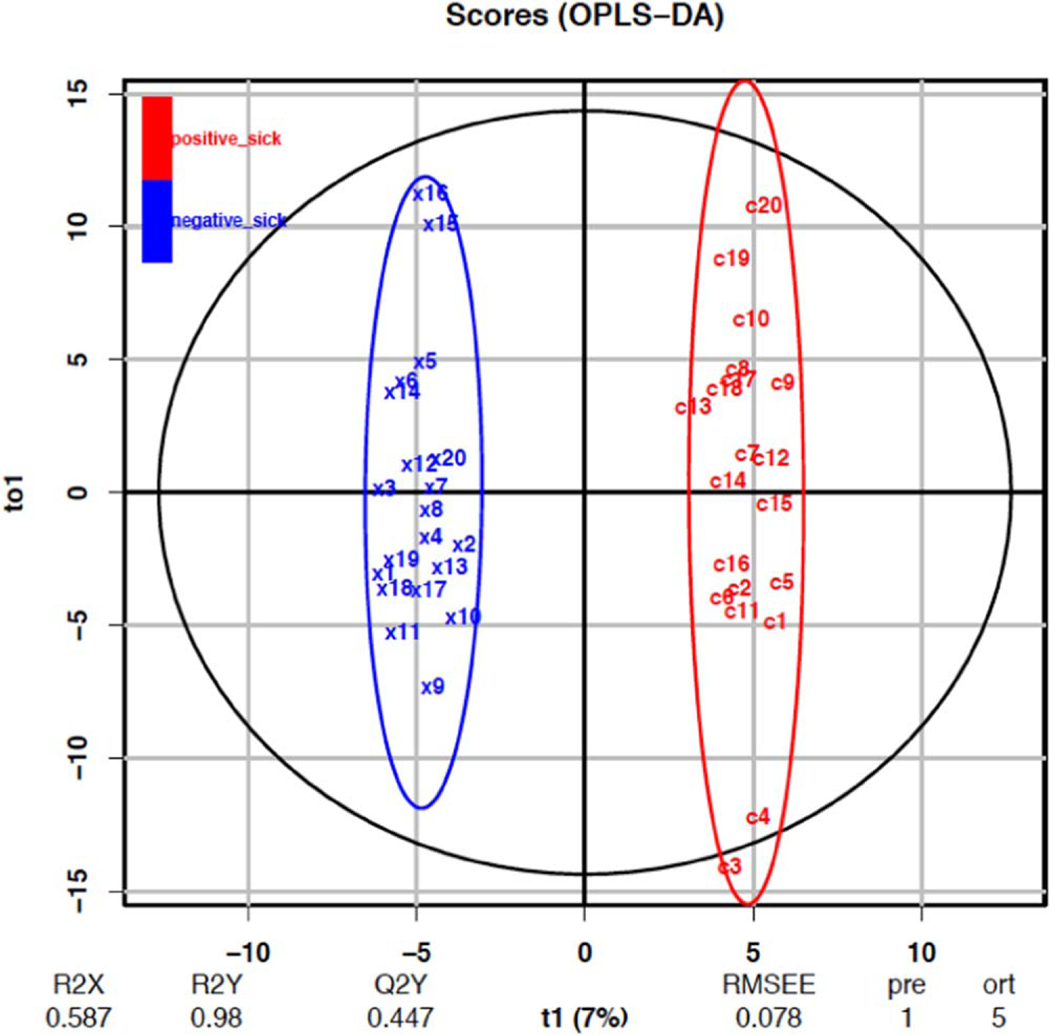
OPLS-DA classification from model using 345 LC-HR-MS features.pR2 = 0.05, pQ2Y = 0.002 from 1000 bootstrap replicates. T1 is the predictive component and TO1 is the first orthogonal component (of5). ‘Positive_sick’ is the case group and ‘negative_sick’ is the control group.

Figure 4 shows the two major-axes plot for the LC-HR-MS results. Here we see separation for samples and replicates using the 345 features. The LC classification model (figure 4, pR2 = 0.05, pQ2Y = 0.002) was able to successfully separate the dog-classified sample groups, with the descriptive component accounting for only 7% of the total feature variance. This model can be interpreted to successfully classify differences between dog-assigned groups using chemical signals; the low variance of the predictive component however, indicates that the majority of the difference between the two groups was not captured by the applied dog labels.

For PLS models, the *variable importance to projection* (VIP) score ranks the significance of individual chemical features to the overall model based on its contributions to the latent variables (Wold et al 2001). For OPLS-DA models, a similar score based on contribution to *only* the predictive variable, (VIP_pred) can be used to rank order underlying features based on their importance to the classification (Galindo-Prieto et al 2014). The top ~10% largest contributors (36) to the classification model, based on VIP_pred score, were selected for closer manual examination. Of the 36 features selected from the LC-MS model, ten were detected in negative ionization mode (see table S1). The ten *m/z* values detected were determined to be associated with cholesterol sulfate and a series of lipids PE-Cer(d16:1/18:0), SM(d18:1/15:0), PA(14:0/17:0), and Lauric acid, three phospholipid compounds, and one fatty acid. Of the chemical features derived from positive mode, the species belong to one or more polyethylene glycol (PEG) polymeric series. PEG polymers are common in consumer plastics and personal care products and are most likely derived from exogenous sources, from the mask materials, or recent subject exposures, rather than human biology.

Further investigation of the 345 features showed that there were three compound groups near the top of the list ordered by contribution to the OPLS-DA model. These likely contained C, H, O (carbon, hydrogen, and oxygen), C, H, N, O (carbon, hydrogen, nitrogen, and oxygen) and C, H, N, O, P, S (carbon, hydrogen, nitrogen, and oxygen, phosphorous, and sulfur), respectively. All of these are intrinsic to life processes.

- C, H, O group: these compounds fall into the general categories of lipids, carbohydrates, and fatty acids.
- C, H, N, O group: these compounds include proteins, aminoacids, amides, and carboxylicacids.
- C, H, N, O, P, S group: these compounds represent the six most abundant elements in life molecules. These compounds are often found together in a variety of biomolecules involved with enzymes, hormones, and protein chemistry.

We chose the top five compounds of each group in the order of their OPLS-DA significance contribution. Table 1 shows the position in the model, the predicted chemical formula, and the observed neutral mono-isotopic mass.

### Between-compound scale

**Figure 5.**
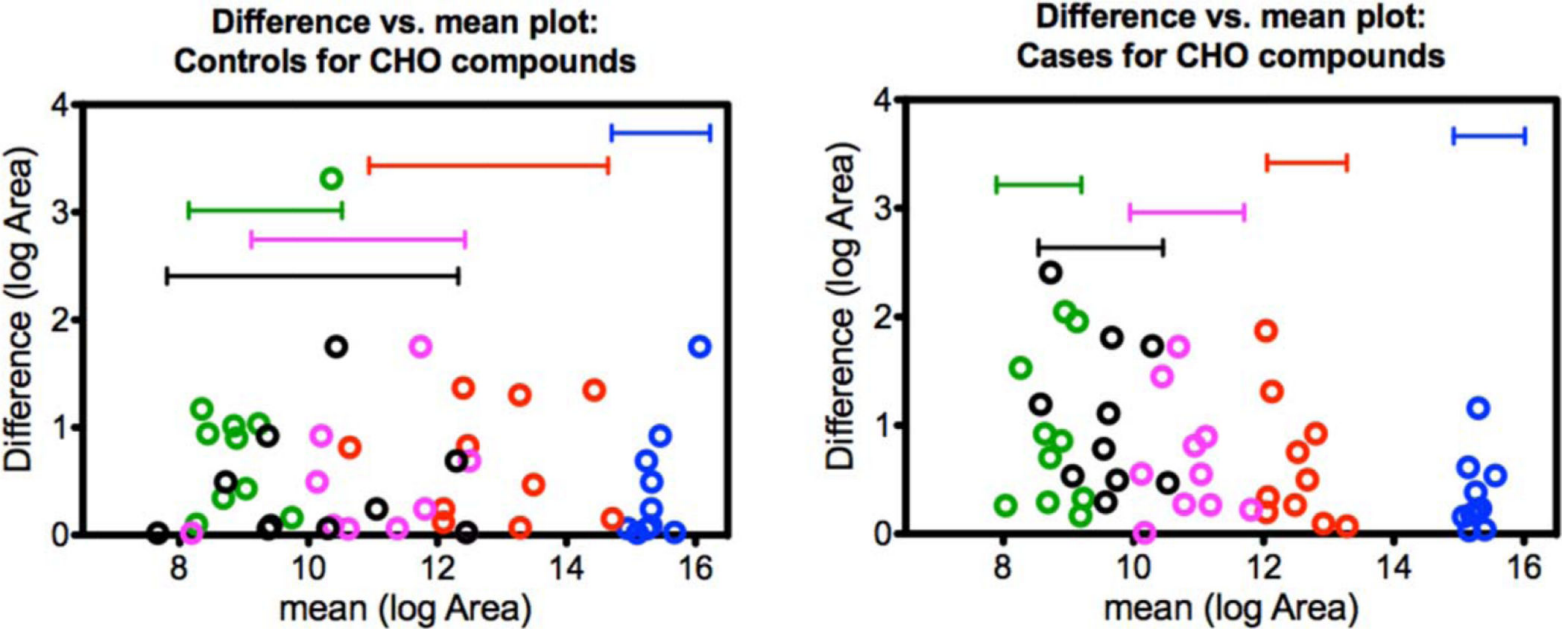
C, H, O compound patterns for controls (left) and cases (right). The values plotted on *x*- and *y*-axes were natural log-transformed (log_e_). Case samples show reduced variability compared to control samples although the area counts of the cases fall within the same ranges as the controls.

**Figure 6.**
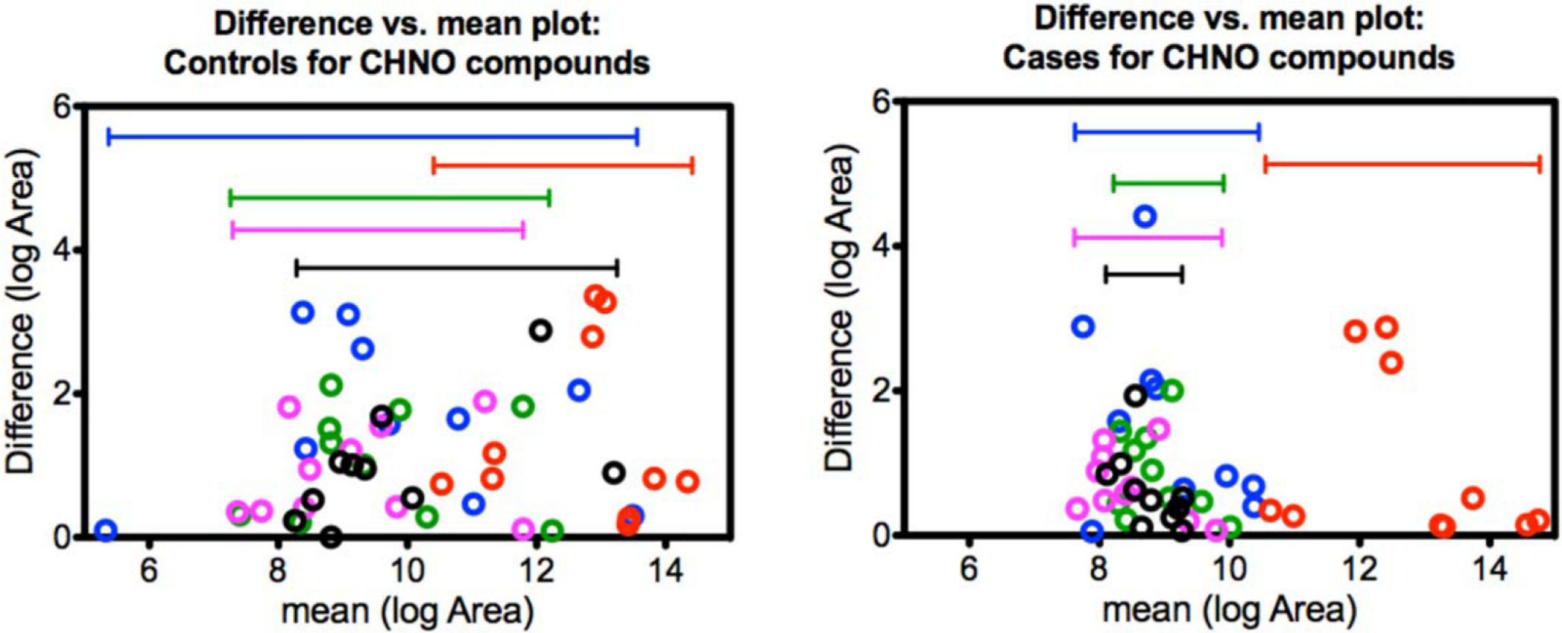
C, H, N, O compound patterns for controls (left) and cases (right). The values plotted on the *x*- and *y*-axes were natural log-transformed (log_e_). Similarly to the C, H, O group in figure 2, case samples show reduced variability compared to control samples although the area counts of the cases fall within the same ranges as the controls.

**Figure 7.**
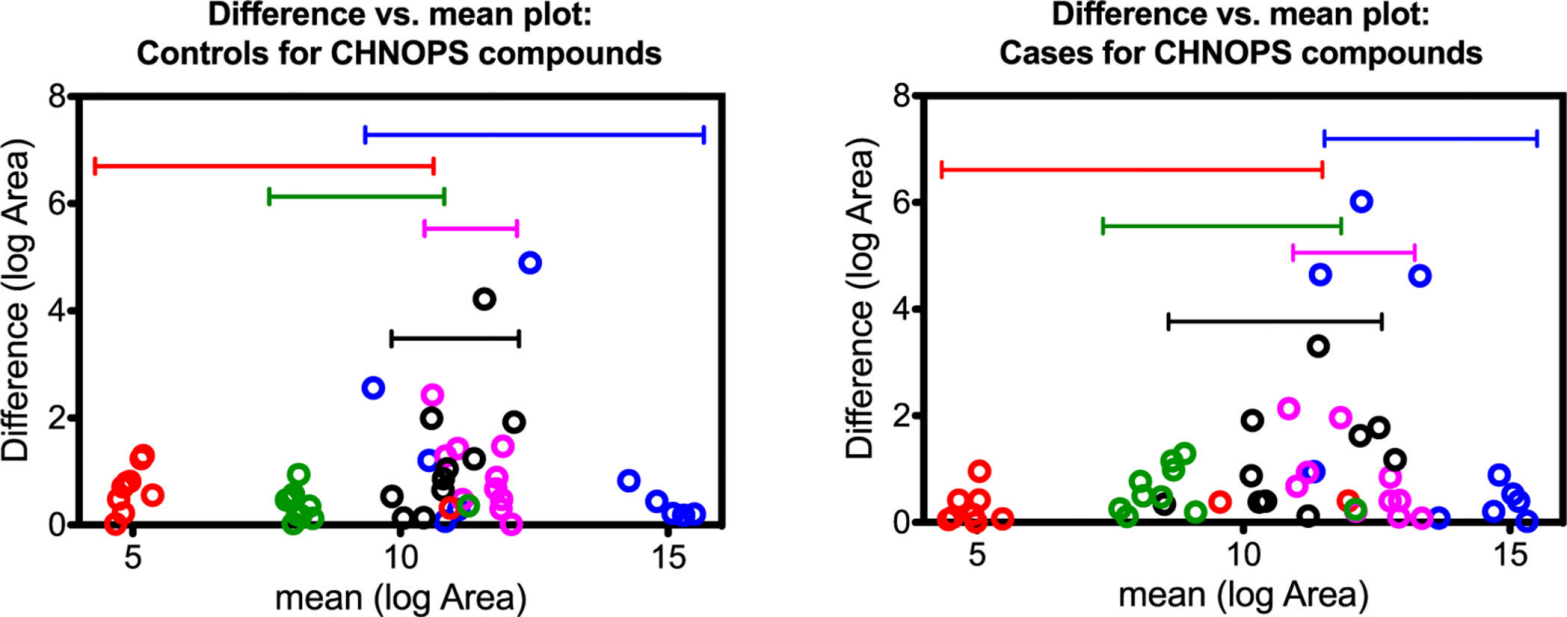
C, H, N, O, P, S compound patterns for controls (left) and cases (right). The values plotted on the *x*- and *y*-axes were natural log-transformed (log_e_). There does not seem to be an apparent pattern difference for this compound class.

Although all features contributed to the OPLS-DA model, the features listed in table 1 were chosen based on similarities in their predicted chemical formulas. Figures 5–7 visualize variability between controls and cases for each of the three groups. The vertical axes indicate within-sample variability (from repeat analyses), and the horizontal axes indicate between-compound scale. Each compound from the respective group is color-coded in order as blue, red, purple, black, and green as indicated in table 1. Horizontal range lines are inserted in each figure panel to guide the eye. Although this is difficult to explain mathematically, the patterns in the control versus case panels appear different for the C, H, O and C, H, N, O groups, but are indistinguishable for the C, H, N, O, P, S group. Such patterns may be one clue as to how the dogs process case-control information. In these graphs, the individual sample is not tracked, only the character of the variances for compounds.

### Within-sample compound variability

A second pattern visualization technique is based on within-sample variability wherein the pattern is based on how compounds track to each other inside individual samples. We have recognized this effect in prior work investigating exogenous exposures and cytokines response. Within-sample patterns were explored using heatmapping and intraclass correlation coefficients in previous articles (Pleil et al 2011a, Stiegel et al 2015, Pleil et al 2018).

**Figure 8.**
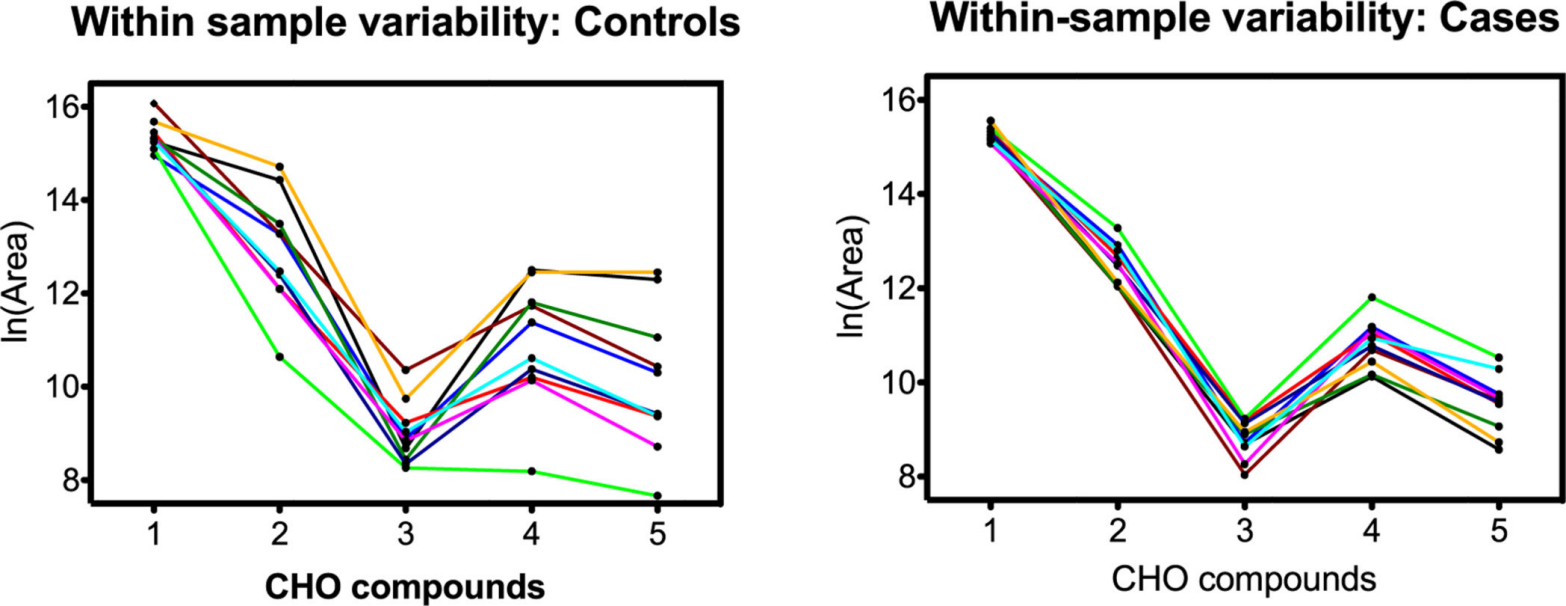
C, H, O compound group comparison of controls and cases. The *x*-axis identifies the five C, H, O compounds in order from table 1; lines connect individual samples. Graphs show more consistent patterns across compounds for cases than for controls.

**Figure 9.**
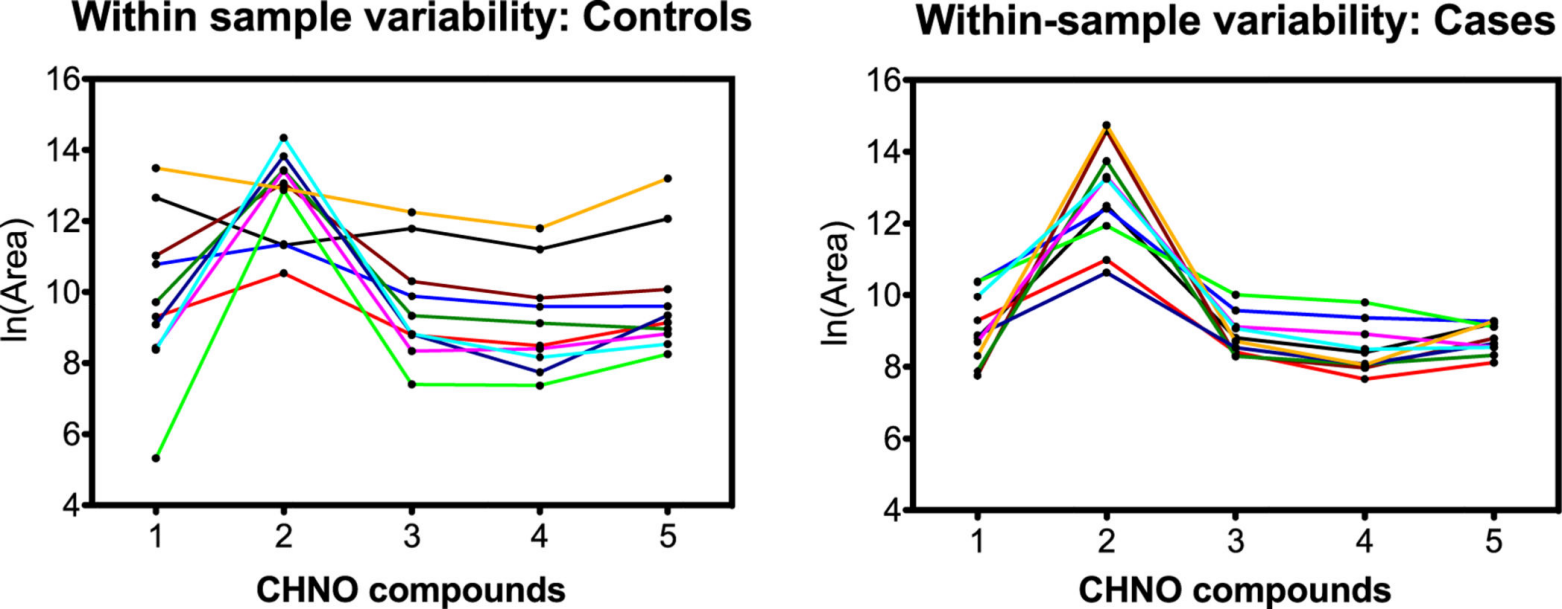
C, H, N, O compound group comparison of controls and cases. The *x*-axis identifies the five C, H, N, O compounds in order from table 1; lines connect individual samples. Graphs show more consistent patterns across compounds for cases than for controls.

**Figure 10.**
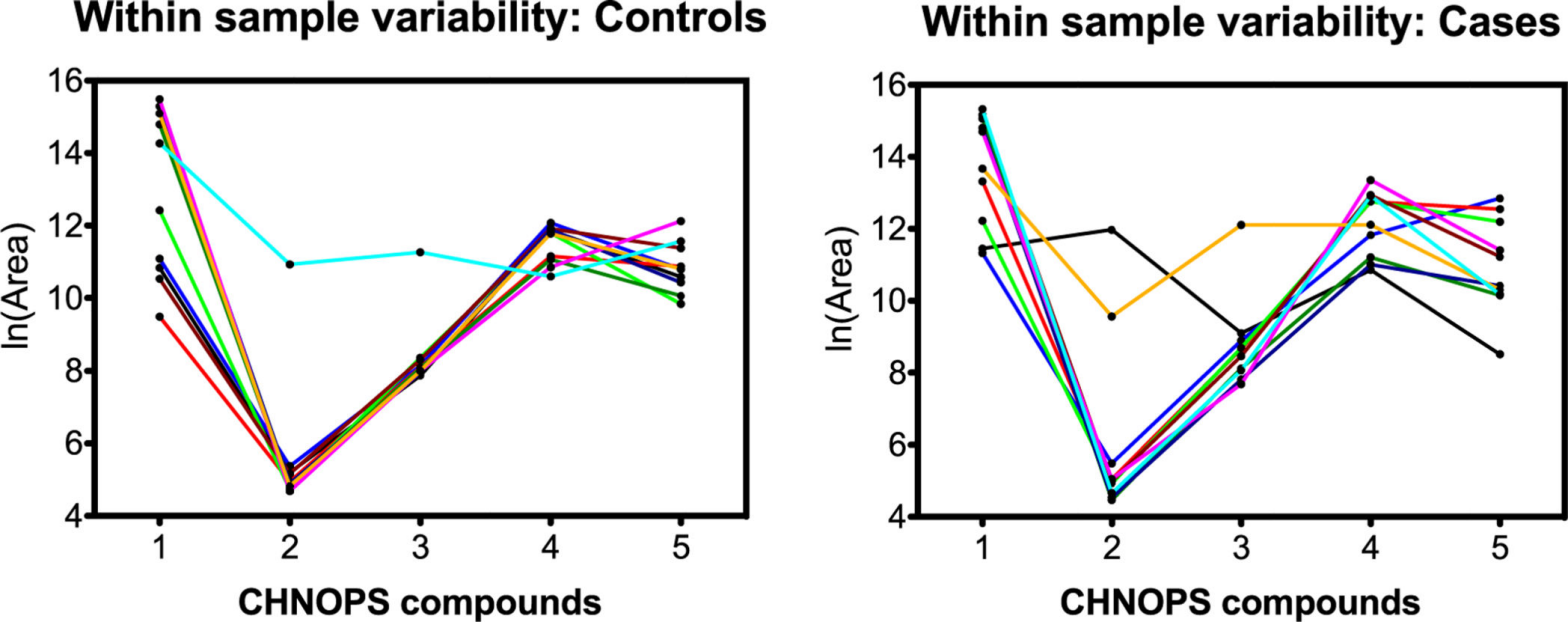
C, H, N, O, P, S compound group comparison of controls and cases. The *x*-axis identifies the five C, H, N, O, P, S compounds in order from table 1; lines connect individual samples. In contrast to previous examples, with the exception of one sample in the controls panel (left), the controls show more consistent patterns across compounds than the cases, especially for compounds 4 and 5.

From a pattern recognition perspective, perhaps it is more important that compounds track each other within a sample, that is, compound A is always higher than B, is higher than C, etc regardless of the total scale as in the section before. This was explored by connecting samples on the horizontal scale jumping from compound to compound. Figures 8–10 are parallel to figures 5–7 with respect to compound group as defined in table 1.

Herein, patterns distinguish controls from cases dependent on within-sample variability. In the C, H, O and C, H, N, O groups, the connecting lines for samples are less likely to cross for cases, indicating more internal consistency in compound pattern; oddly, the C, H, N, O, P, S group exhibits the inverse result. Regardless, within-sample variability is different between controls and cases, and this may contribute to the dogs’ ability to sort.

### Summary of LC-HR-MS results

The OPLS-DA model of 345 features was able to separate the control and case mask samples; the descriptive component of the model, however, only accounted for 7% of the total feature variance (figure 4). Further detailed analyses showed that sets of non-volatile compounds demonstrate slight pattern differentiation when analyzed for between-compound scale (figures 5–7) and also for within-sample variability (figures 8–10). It is likely that the odor patterns detected by the dogs have at least some overlap with the semi- and non-VOCs found in the LC-HR-MS analysis. However, the authors recognize that there are likely many probative compounds that the dogs are able to detect that were not captured in the laboratory analyses in this study.

## GC-MS results

Although the GC-MS data have better chromatographic resolution than LC, the lower specificity from the unit Da resolution made specific feature identifications more difficult. Nevertheless, 44 candidate features were annotated by retention time and prominent mass fragments, and then used for OPLS-DA modeling. Of these, 26 were assigned a likely chemical formula and tentative name based on library search (see supplemental table S2).

### Feature-based OPLS-DA

When the OPLS-DA approach was applied to the GC-MS results, the program calculated the model using 44 detected features, as shown in figure S1 in section 2 of the supplemental information. This analysis showed some visual separation of the case and control samples; however, the GC classification model was not able to produce a statistically significant model (figure S1, pR2 = 0.45, PQ2Y = 0.80). This should be interpreted as an indication that the chemical signals captured by the GC-MS preparation and analysis were insufficient to unambiguously replicate the case-control assignment of the trained dogs.

Of interest in the GC-MS analyses were the types of chemical formulas predicted and their relatively lower masses compared to those from the LC-HR-MS analyses. Upon further investigation of the OPLS-DA analysis of the GC-MS data, a group of five hydrocarbon (C, H) compounds and a group of four C, H, O compounds were selected as likely to be endogenous based on predicted chemical formula (table 2). Specifically, table 2 shows the predicted chemical formulas, which are based on single Da mass spectral library searches. Each feature is discreet as they all have different GC retention times, despite apparent similarities in predicted chemical formulas. Note that the C_8_H_18_ (position 9) entry identification is likely based on a fragment of a larger C, H compound closer to 180 Da based on retention time.

As these are features found in GC-MS, their volatility is higher than those detected by LC-HR-MS, and their respective integer masses are lower than those of the features found in LC-HR-MS. This reduces the probability of these features being useful for case-control analysis as these compounds were extracted from masks, and thus were more susceptible to evaporative losses between sampling and analysis.

Data reduction for the GC analytes showed a relatively constant range across all compounds; as such the between-compound scale analysis as shown for the LC-HR-MS analyses in figures 5–7 did not demonstrate useful patterns. However, the within-sample compound variability analysis seemed to show some pattern differences for the C, H compounds.

### Within-sample compound variability

**Figure 11.**
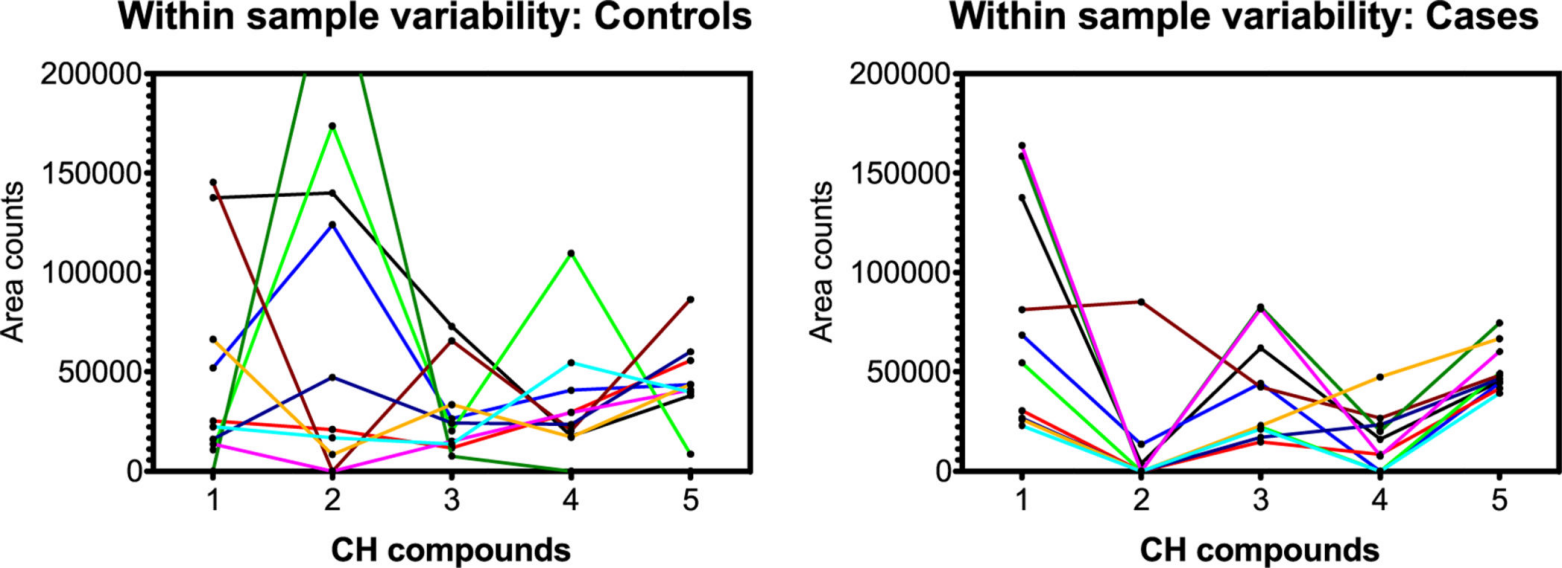
C, H compound group comparison of controls and cases. The *x*-axis identifies the five C, H compounds in order from table 2 with ‘Y’ designators; lines connect individual samples. Graphs indicate more within-sample (i.e. between-compound) and within-compound variability for controls than for cases.

**Figure 12.**
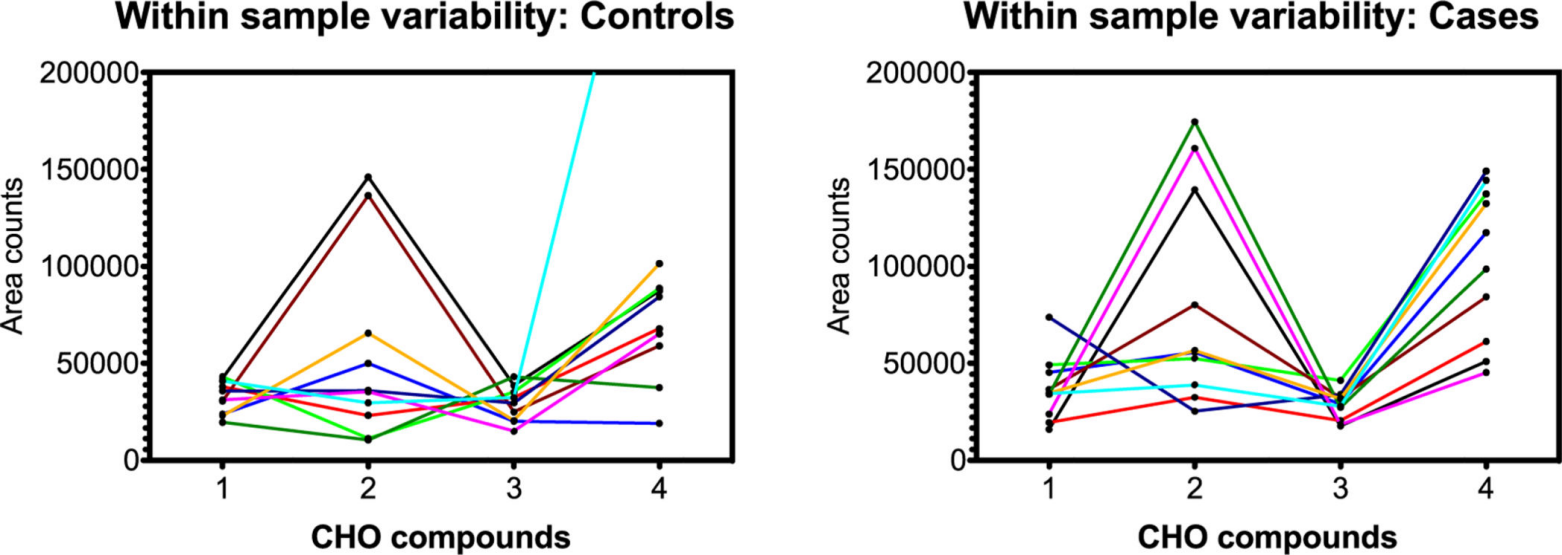
C, H, O compound group comparison of controls and cases. The *x*-axis identifies the four C, H, O compounds in order from table 2; lines connect individual samples. Graphs indicate no obvious differences for within-sample (i.e. between-compound) and within-compound variability for cases versus controls.

As in the LC-HR-MS section, only certain compounds were chosen for graphing. Figures 11 and 12 show the within-sample variability for five selected C, H compounds and four C, H, O compound groups from table 2.

### Summary of GC-MS results

The OPLS-DA model developed using the 44 distinct GC-MS features was unable to separate the controls and cases with any degree of confidence. Further detailed analyses on one set of selected C, H compounds demonstrates some level of pattern differentiation when analyzed for within-sample (i.e. between-compound) variability (figure 11). The C, H, O compounds (figure 12), however, do not show any obvious discrimination. This suggests that the detectable GC-MS compounds are not significant contributors to the dog classification. The mask sampling approach was not expected to capture substantial quantities of GC-able semi-volatiles, so this result is somewhat expected.

## Conclusions

This work is exploratory and was conducted with a limited sample set of masks that were unambiguously sorted as case or control by four dogs independently. While it is unknown whether or not the dogs were correct in their sample classifications, there is some evidence (based mostly on LC-HR-MS results) that the two groups of samples may be different analytically, although more experiments are needed to confirm this finding. This research illuminates fundamental questions about pre-clinical disease screening methods, leading to the following observations:

- Dogs are capable of sorting biological samples based on exhaled aerosols captured on standard hospital masks; they do not necessarily require gas-phase metabolites.
- Immunochemistry analysis for human cytokines demonstrated that exhaled aerosols are found on 2 ml extracts of filter sections and detected at or below 0.1 pg ml^−1^ implying that other detected organic molecules also have human origins.
- LC-HR-MS analysis detected 345 high quality organic features on masks.
- Subsets of selected LC-HR-MS features demonstrated recognizable case-control differences based on patterns observed between and within samples.
- GC-MS analysis detected fewer high quality organic features (44) on masks. This may be due to low retention of volatile compounds on mask samples.
- OPLS-DA models replicating the dog classifications were constructed using LC-MS and GC-MS data, but were only statistically significant for LC-MS data.

The two most important findings from this work are:

- Evidence suggests that low-volatility organic compounds in exhaled breath aerosol can be used to sort biological samples into distinct groups.
- There may be chemical-space overlap in organic compounds detected by canine olfaction and analytical instrumentation.

Breath aerosols that are collected on disposable masks serve as a probative biological medium for NTA via LC-HR-MS. While these results may include potential confounding from inhalation of exogenous compounds that were captured by the masks, this does not affect the overall design of this study since these compounds should have also been detected by the dogs. Future work will include obtaining larger subsets of case and control samples from CancerDogs and repeating the non-targeted LC-HR-MS analyses. Replicate mask samples that are prepared can be extracted using multiple techniques to increase the number and types of compounds that are detected. With a larger sample set, we hope to create a stronger model that may be predictive for determining whether an unknown, blinded sample is from the case or control group.

## Supplementary Material

Sup1

Sup2

## Figures and Tables

**Table 1.** Groups of features contributing to OPLS-DA analysis. ‘OPLS-DA order’ indicates rank of importance amongst all 345 modeled features; ‘predicted chemical formula’ was calculated from HR-MS, isotope patterns, and fractionation; ‘observed neutral monoisotopic mass’ was assigned by the Orbitrap instrument. Color code refers to figures 5–7.

OPLS-DA order (of 345)	Predicted chemical formula	Observed neutral monoisotopic mass (Da)	Retention time (min)	Color code (see figures 5–7)

8	C22H44O6	404.314 13	14.455	Blue
10	C20H42O5	362.302 82	11.452	Red
12	C26H52O6	460.375 93	17.269	Purple
19	C18H38O5	334.271 49	9.289	Black
24	C19H40O4	332.292 26	12.708	Green
2	C20H43NO5	377.313 75	8.981	Blue
3	C23H45N5O8	519.325 35	1.698	Red
4	C22H43N5O2	409.340 01	10.335	Purple
9	C21H41N5O2	395.324 26	9.348	Black
11	C27H53N5O3	495.413 40	13.133	Green
5	C83H163N4O15P3S	1581.105 20	17.228	Blue
9	C78H147N8O13P3S	1529.002 29	9.348	Red
26	C47H105N10O8P3S2	1094.676 85	2.062	Purple
27	C55H122N8O17P2S	1260.812 85	9.292	Black
31	C90H163N8O17PS2	1723.131 88	14.911	Green

**Table 2.** Tentative identifications of selected features from GC-MS analyses based on library comparisons of single Da resolution mass spectra.

OPLSDA order (of44)	Predicted chemical formula	Integer mass (Da)	Retention time(min)	Compound number(in figures 11– 12)

1	C10H22	142	11.126	1
8	C13H28	184	21.157	2
9	C8H18	114	21.733	3
10	C10H22	142	11.667	4
43	C10H22	142	18.662	5
2	C7H14O2	130	15.770	1
36	C6H12O	100	11.129	2
42	C7H16O	116	15.922	3
44	C4H8O	72	5.995	4
