# Supplementary material for: How do cancer-sniffing dogs sort biological samples? Exploring case-control samples with non-targeted LC-Orbitrap, GC-MS, and immunochemistry methods
Source: J Breath Res. Author manuscript; Available in PMC 2021 Dec 7. (PMC8649743; doi:10.1088/1752-7163/ab433a)
Supplement: Sup1 [file NIHMS1544864-supplement-Sup1.pdf]

Supplemental Information

How do cancer-sniffing dogs sort biological samples? Exploring case-control samples with non-targeted LC-Orbitrap, GC-MS and immunochemistry methods

1. LC-MS Supplemental Sample Analysis Methods

Sub-aliquots of 100 µL volume were removed from the MS designated samples in methanol and diluted with 300 µL of 2 mM ammonium formate. Sample order was randomized prior to analysis and two injection replicates were performed for each prepared sample. Double blank samples consisting of 100 µL laboratory MeOH and 300 µL of 2mM ammonium formate were analyzed every five injections to check for substantive carryover between samples. A 25 µL injection of the diluted samples was separated on a Vanquish UPLC (Thermo Scientific) system equipped with an Accucore Vanquish C18+ column (100 x 2.1 mm, 1.5 µm particle diameter) coupled to an Orbitrap Fusion (Thermo Electron) for data collection. The chromatographic gradient consisted of a single linear gradient from 25-100% B over 18 min, with mobile phases A: 95/5 H_2O/MeOH (0.1% formic acid) and B: 95/5 MeOH/H_2O (0.1% formic acid) and a flow rate of 300 µL/min. Columns were flushed for 3 min with 100% B after each sample and equilibrated with 25% B prior to the next injection.

MS data were collected with both positive and negative electrospray ionization in data-dependent MS/MS mode with a 0.6 s cycle time. Electrospray ionization used the HESI source and conditions were, in positive mode: 3.0 kV spray voltage, sheath gas 11 (arb), aux gas 4 (arb), sweep gas 0 (arb), ion transfer tube 350 °C, vaporizer temperature 275 °C. MS1 data were collected at 120,000 nominal resolving power, RF lens 45, automatic gain control (AGC) target 5e5, maximum injection time 250 ms, and a mass range of 150-1500 Da.

Data-dependent acquisition was based on monoisotopic precursor selection using a small molecule model, with threshold abundance of 2e4 and a 6 s dynamic exclusion after MS/MS selection.

MS/MS data were collected at 15,000 nominal resolution in the orbitrap analyzer with a 1.6 Da quadrupole isolation window, stepped HCD collision energy of 35 +/- 1, fixed first mass of 80 Da. The AGC target was 5e4 with ions injected for all maximum parallelizable time (22 ms).

2. GC-MS Supplemental Data

A total of 44 detected features were used to build the OPLS-DA model, as shown in Figure S1. The GC classification model was not statistically significant (pR2 = 0.45, PQ2Y = 0.80). This strengthens the need for a model validation processes (Wehrens 2011) and is an example of potential over-modeling, sometimes referred to as “voodoo correlation”, which is an effect that is identified by randomizing the training set repeatedly and observing similar results.

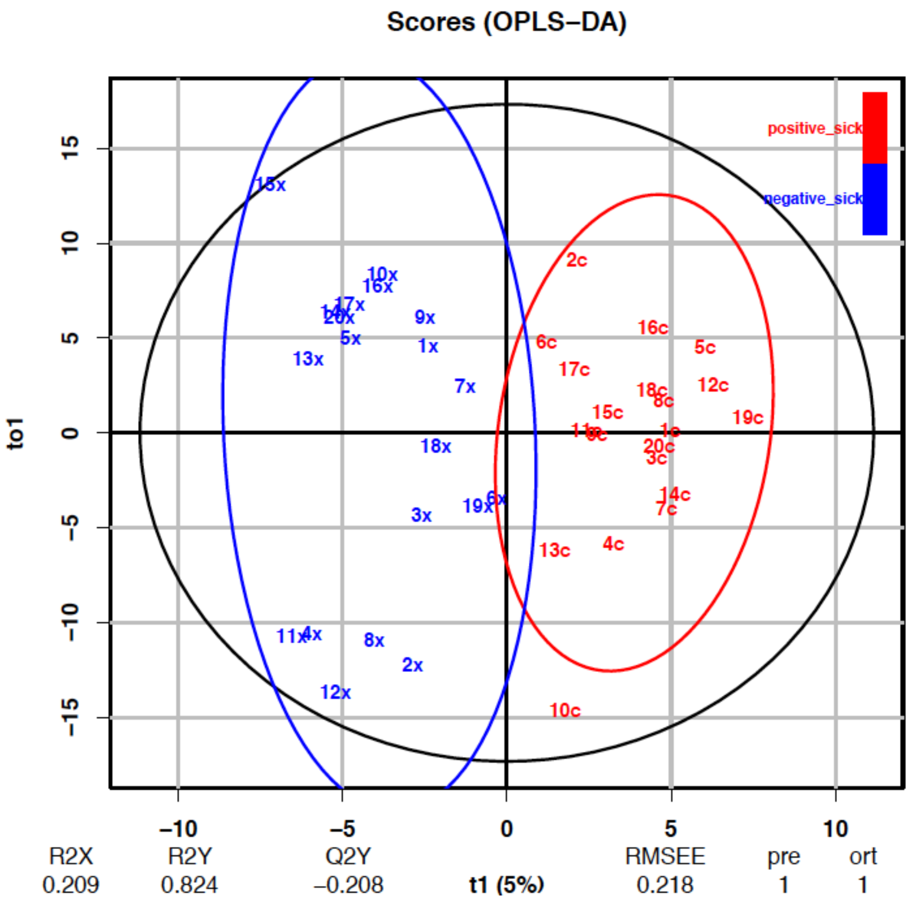

Figure S1: OPLS-DA classification model using 44 GC-MS features. pR2 = 0.45, PQ2Y = 0.80 from 1000 bootstrap replicates. T1 is the predictive component and TO1 is the orthogonal component. Positive_sick (red c) are the cases and negative_sick (blue x) are the control samples.
